# Petri-Net-Based Charging Scheduling Optimization in Rechargeable Sensor Networks

**DOI:** 10.3390/s24196316

**Published:** 2024-09-29

**Authors:** Huaiyu Qin, Wei Ding, Lei Xu, Chenzhi Ruan

**Affiliations:** 1 School of Electrical and Information Engineering, Jiangsu University of Science and Technology, Zhenjiang 212000, China; dingwei_just@just.edu.cn (W.D.); emmaleilei@just.edu.cn (L.X.); 2 The Key Laboratory for Agricultural Machinery Intelligent Control and Manufacturing of Fujian Education Institution, Wuyishan 354330, China; ruanczhi@wuyiu.edu.cn; 3 College of Mechanical and Electrical Engineering, Wuyi University, Wuyishan 354330, China

**Keywords:** wireless rechargeable sensor network, charging benefit, Petri net, ant colony algorithm, precision agriculture

## Abstract

In order to express the energy flow, motion flow, and control flow in wireless rechargeable sensor networks accurately and intuitively, and to maximize the charging benefit of MVs (mobile vehicles), a type of MTS-HACO (Mobile Transition Sequence Hybrid Ant Colony Optimization) is proposed. Firstly, node places are grouped according to the firing time of node’s energy consumption transition to ensure that in each time slot, MV places only enable charging transitions for the node places with lower remaining lifetimes. Then, the FSOMCT (Firing Sequence Optimization of Mobile Charging Transition) problem is formulated under the constraints of MV places capacity, travelling arc weight, charging arc weight, and so on. The elite strategy and the Max–Min Ant Colony system are further introduced to improve the ant colony algorithm, while the improved FWA (fireworks algorithm) optimizes the path constructed by each ant. Finally, the optimal mobile charging transition firing sequence and charging times are obtained, ensuring that MVs have sufficient energy to return to the base station. Simulation results indicate that, compared with the periodic algorithm and the PE-FWA algorithm, the proposed method can improve charging benefit by approximately 48.7% and 26.3%, respectively.

## 1. Introduction

Precision agriculture is a key direction in the development of modern agriculture. Through the use of sensor technology and other methods, it enables precise monitoring and management of crop growth environments, thereby improving agricultural production efficiency and reducing resource waste. By deploying a large number of sensors in the field, agricultural producers can obtain real-time information on soil moisture, temperature, light intensity, and weather conditions, providing scientific guidance for crop growth. However, the widespread use of these sensors has also introduced new challenges, particularly the energy constraints of sensor batteries. To address this challenge, wireless rechargeable sensor networks (WRSNs) have gained increasing attention in recent years. Moreover, against this backdrop, charging scheduling methods based on Petri nets have garnered increasing attention from researchers due to their advantages in modeling complex systems [1,2].

In most charging strategies, the scheduling methods typically involve either charging all nodes along a fixed trajectory or randomly charging sensor nodes. However, there are still several drawbacks to these strategies.

Most existing strategies base their charging decisions on the remaining energy of the nodes, but they fail to account for differences in node lifespans. A node with low remaining energy but a low energy consumption rate may not necessarily have a short lifespan. Conversely, a node with relatively high remaining energy but a high consumption rate might deplete its energy more quickly. Therefore, these charging strategies do not fully reflect the actual energy needs of the nodes. In most studies, nodes are fully charged. However, in practical scenarios, charging lithium batteries beyond approximately 90% significantly slows down the process, resulting in low time efficiency [3]. The charging benefit during the charger’s movement is often overlooked, yet it directly affects energy replenishment and the network’s condition. Therefore, improving charging benefit is crucial for optimizing energy utilization and enhancing overall network performance.

To overcome the aforementioned shortcomings, it is necessary to determine which nodes should be charged during each time slot and the number of MVs (mobile vehicles) to deploy. Moreover, task allocation and path selection for MVs are crucial for improving charging benefit. Additionally, to accurately describe the energy state, charging behavior, and traveling path within the network, and thereby analyze network behavior and optimize scheduling strategies, this paper proposes a Generalized Synchronizing Continuous Cyber Petri-net System (GSCCPNS). The charging scheduling problem is then formulated as the Firing Sequence Optimization of Mobile Charging Transition (FSOMCT), and the Mobile Transition Sequence Hybrid Ant Colony Optimization (MTS-HACO) is proposed. The main contributions of this paper are as follows:

First, the Generalized Synchronizing Continuous Cyber Petri-net System is innovatively proposed, where place markings represent the real-time energy of MVs (sensor nodes); transitions represent events such as traveling, charging, or energy consuming; and directed arc weights represent charging power or consuming power. This unifies the models of energy flow, motion flow, and control flow in wireless rechargeable sensor networks.

Second, the shortest firing time of node’s energy consumption transition is considered as the slot time. Node places to be charged for each time slot are determined based on the firing time of each node’s energy consumption transition, ensuring that each node receives timely energy replenishment without wasting energy.

Third, by combining an improved ant colony algorithm with the fireworks algorithm, the MTS-HACO algorithm optimizes the mobile charging transition firing sequence. This ensures nodes are replenished with energy in a timely manner while improving the network’s overall charging benefit.

The remaining sections of this paper are organized as follows: Section 2 reviews the latest advancements in charging scheduling optimization and the application of Petri nets. Section 3 establishes the Petri-net model for wireless rechargeable sensor networks. Section 4 formulates the Firing Sequence Optimization of Mobile Charging Transition problem. Section 5 proposes the MTS-HACO algorithm to solve this problem. Section 6 analyzes the simulation results. Finally, Section 7 concludes the paper and discusses future work.

## 2. Related Work

In wireless rechargeable sensor networks, researchers have introduced various charging strategies to address the issue of node lifespan. These strategies can be categorized into fixed charging and mobile charging, based on charger deployment methods, and they primarily involve optimization through intelligent algorithms and deep learning techniques.

### 2.1. Charging Scheduling Optimization

Due to the inherent limitation of fixed charging, which cannot fully cover all areas, we first proposed the master-slave charging strategy, with fixed charging as the primary method to cover most sensor nodes, supplemented by mobile chargers to serve the weakly covered areas of fixed chargers [1]. Building on this, Deng et al. proposed a hybrid charging scheme that combined fixed and mobile chargers to minimize the number of energy-depleted nodes [4]. Xu et al. studied multi-node energy charging schemes with multiple mobile chargers (MCs), optimizing the charging schedule to minimize the maximum charging delay [5]. Yang et al. proposed a multi-type charging scheduling scheme based on regional demand differences, using different topological structures in the inner and outer rings to improve node survival rates and energy utilization efficiency [6].

Optimization of scheduling strategies primarily leverages intelligent algorithms and reinforcement learning techniques to enhance charging schedules, thereby improving network energy efficiency and node survival rates. Hu et al. proposed a periodic scheduling algorithm to balance charging tasks within predefined time slots, effectively preventing energy starvation caused by charging delays [7]. Lyu et al. introduced a multi-node charging and data collection scheme based on mobile devices, optimizing anchor point locations and path planning to maintain perpetual network operation [8]. Sha et al. presented a periodic distributed energy replenishment method based on maximizing charging efficiency, optimizing the charging efficiency of wireless sensor networks [9]. Li et al. studied a multi-objective optimization charging scheme aimed at extending the network lifetime of wireless rechargeable sensor networks [10]. Dash (2023) explored a two-stage energy-saving load balancing scheme to improve data collection efficiency in energy-harvesting wireless sensor networks, utilizing mobile sink nodes for data collection and applying minimum-cost network flow techniques to optimize data distribution [11]. Although this method improves data collection throughput, its reliance on mobile sink path planning may result in additional communication overhead. Guo et al. investigated the adaptive profit balancing between multiple mobile wireless chargers to optimize overall charging utility [12].

Jiang et al. proposed a joint sequence-scheduling and trajectory-planning scheme using deep reinforcement learning, enabling mobile chargers to avoid obstacles and optimize charging performance [13]. Guo et al. introduced an adaptive dual-mode energy-efficient routing scheme based on deep Q-networks to enhance the sustainability of rechargeable wireless sensor networks [14]. Shan et al. proposed a multi-UAV path planning scheme based on deep reinforcement learning to optimize the charging paths in wireless rechargeable sensor networks [15].

Although deep learning techniques have achieved some success in optimizing scheduling in WRSNs, they have limitations in terms of data requirements, training time, model generalization, and interpretability. In contrast, intelligent algorithms such as genetic algorithms, particle swarm optimization, and ant colony algorithms demonstrate good adaptability and computational efficiency in optimization problems, making them well suited to meeting scheduling demands in dynamic network environments. Therefore, this paper proposes a scheduling strategy based on an improved ant colony algorithm, incorporating an elite strategy and the fireworks algorithm for local optimization, to obtain the optimal mobile charging transition firing sequence.

### 2.2. Petri-Net Application

As a powerful modeling tool, Petri nets have been widely applied in resource allocation, scheduling optimization, and system verification. Existing research can be broadly categorized into the following areas: system modeling and verification, liveness analysis, and scheduling optimization.

Petri nets possess strong expressive capabilities in system modeling and verification, effectively handling complex system behaviors and state transitions. Yang et al. proposed a real-time mask recognition system based on Petri nets, using the Jetson Nano edge computing platform and YOLOv5 model for automatic mask recognition, and employed the Workflow Petri Net Designer for system verification [16]. Zhong et al. introduced a time-triggered network verification framework based on timed colored Petri nets, ensuring real-time communication requirements through automated modeling and reachability analysis [17]. Hu et al. explored the optimal sensor selection problem for diagnostic enforcement in discrete event systems based on Petri nets, optimizing system diagnostics using nondeterministic finite automata and integer linear programming [18].

Liveness analysis is a crucial method for ensuring that systems run without deadlocks. Qi et al. proposed a liveness decision method from a machine learning perspective, using deep neural networks to learn feature information of unbounded Petri nets and probabilistically predict their liveness [19]. Yue et al. introduced an algorithm for estimating the minimum initial markings of marked Petri nets with unobservable transitions, optimizing resource allocation through minimal interpretation [20]. Dou et al. presented a novel Petri-net liveness analysis method based on maximum good step graphs, which significantly reduces state space and time consumption in liveness analysis [21].

Petri nets also have significant applications in resource allocation and scheduling optimization. Lv and Huang proposed an arbitrary-time A* search method for scheduling in Petri-net-based resource allocation systems, combining A* search with depth-first search to improve scheduling efficiency [22]. Ahn and Kim introduced a novel mixed-integer programming model for non-periodic integrated tool scheduling, using timed Petri nets and priority relations to reduce decision variables and enhance scheduling performance [23]. Hustiu et al. developed a multi-robot path planning algorithm using Petri nets and linear temporal logic specifications, which reduced computational complexity [24]. In a previous work, we proposed a hybrid colored cyber net system for multi-UAV scenarios, performing multi-objective optimization of wireless power and information transmission networks, achieving superior performance compared with NSGA-II and MOEA/D [1].

Despite significant progress in system modeling, scheduling optimization, and liveness analysis using Petri nets, existing methods still face challenges in adapting to dynamic network changes, computational complexity, and practical applications. In particular, effectively utilizing Petri nets for optimizing scheduling in WRSNs to meet evolving network demands and resource constraints remains a pressing challenge. Building on existing research, this paper introduces a novel WRSN optimization scheduling method that integrates advanced intelligent algorithms with the powerful modeling capabilities of Petri nets. The following section provides a detailed description of the modeling approaches for the Petri-net model of WRSNs.

## 3. Preliminaries

In this section, the model of the wireless rechargeable sensor network is first presented. Then, based on the classical Petri net, the Generalized Synchronizing Continuous Cyber Petri Net System (GSCCPNS) is proposed, and the GSCCPNS model for a WRSN is established.

**Figure 1:**
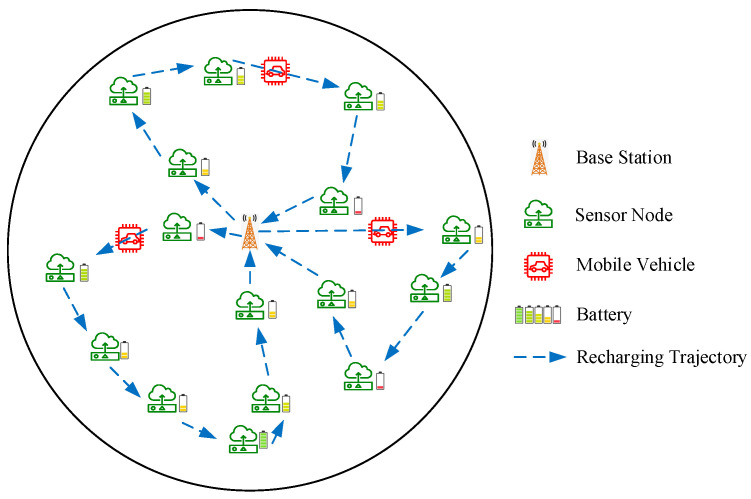
Model of wireless rechargeable sensor network.

In a two-dimensional area *D*, *n* nodes are randomly deployed, with $s_{i}$ representing the *i*th node, $S=\left\{s_{1},\cdots ,s_{i},\cdots ,s_{n}\right\}$, i∈[1,n], i∈ℤ. Simultaneously, *m* mobile vehicles (MVs) are employed, with $c_{j}$ representing the *j*th MV, $C=\left\{c_{1},\cdots ,c_{j},\cdots ,c_{m}\right\}$, j∈[1,m], j∈ℤ. Each MV is responsible for charging a subset of nodes, and each node can be charged by only a single MV within each time slot, ensuring that the sets of charging nodes do not overlap. Figure 1 illustrates the wireless rechargeable sensor network model. For simplicity, it is assumed that the base station (BS) is located at the center of the network, and nodes transmit data to it via multi-hop communication. Additionally, this study employed magnetic coupling for wireless charging, requiring the MVs to be in close proximity to the sensors for recharging. Thus, the positions where the MVs stop are considered to coincide with the positions of the sensor nodes.

Petri nets are a graphical and mathematical modeling tool that provide an intuitive and precise way to characterize complex dynamic behaviors and interactions between components within a system. By constructing a Petri-net model, the charging scheduling process in WRSNs can be effectively described, allowing the optimization of charging strategies and thereby improving the overall energy efficiency and reliability of the network. This paper extends the classical Petri net by proposing the GSCCPNS, which is defined as follows.

**Definition** **1.** *An eight-tuple ∑=(S,T;F,W,M,Inhibit,E,K) is called a Generalized Synchronizing Continuous Cyber Petri-Net System (GSCCPNS) if the following conditions are satisfied:*

*(1)*  *N=(S,T;F) is a directed net, which is the base net of ∑;*
*(2)*  *F⊆S×T∪T×S and Inhibit represent flow relationships and constraint relationships, respectively;*
*(3)*  *$K=\left\{k_{L},k_{H}\right\}$ is the capacity function’s upper and lower bounds, and $k_{L}:S\to \mathbb{R}$, $k_{H}:S\to \mathbb{R}$, ℝ is the set of real numbers;*
*(4)*  *W:F→ℝ∪Exp(S,τ) is the weight function, and Exp(S,τ) is the set of functional expressions of time τ;*
*(5)*  *M:F→ℝ is the marking of place, and $M_{0}$ is the initial marking;*
*(6)*  *$E=\left\{e_{1},e_{2},\cdots \cdots \right\}$ is the event set of ∑.*

**Definition** **2.** *The condition for the firing of transition T is:*

∀s∈T·:(M(s)−WM(s,T))∈[kL(s),kH(s)]∧∀s∈T·:(M(s)+WM(T,s))∈[kL(s),kH(s)]∧∀s∈TW·:W(T,s)∈[kL(s),kH(s)]

*If transition T is fired, it can be denoted as M[T>, where T· and $T^{\cdot }$ are the input and output places of transition T, respectively.*

**Definition** **3.** *Result of Transition Firing: If transition T fires, the original marking M changes to $M^{\prime }$, such that:*

(1) M(s)′={M(s)−W(s,T)s∈T·−T·M(s)+W(T,s)s∈T·−T·M(s)−W(s,T)+W(T,s)s∈T·∩T·W(T,s)s∈TW·M(s)s∉(T·∩T·∪TW·)

The dynamic equation of GSCCPNS is given by:

(2) $$M^{\prime }=M_{0}+\to C\cdot U=M_{0}+C\cdot \left[\int_{\tau_{0}}^{\tau_{n}}\left(\right)\right]d\tau$$

where the matrix operator +→ represents the substitution addition, C is the incidence matrix, and U is the concurrent step sequence matrix.

To apply Petri nets for WRSN modeling, the network elements need to be mapped to places, transitions, and directed arcs. Places represent the real-time energy levels of MVs (sensor nodes), with the capacity function’s upper and lower bounds representing the battery capacity and minimum energy levels of nodes or MVs, respectively. Transitions represent events such as traveling, charging, or energy consumption. Directed arc weights represent charging power or consumption power. A complete charging cycle is divided into several charging time slots, with each slot having a duration of $T_{0}$. In each time slot, all MVs first stay at the base station for a certain period. Then, they leave the base station to charge the required nodes along their movement trajectories and finally return to the base station at the end of the time slot. The variable *v* represents the moving speed of the chargers, and δ represents the energy consumed by the MV per second. After charging all nodes along their trajectory, the MVs should retain enough energy to return to the base station. The definitions of the network model parameters are provided in Table 1.

**Figure 2:**
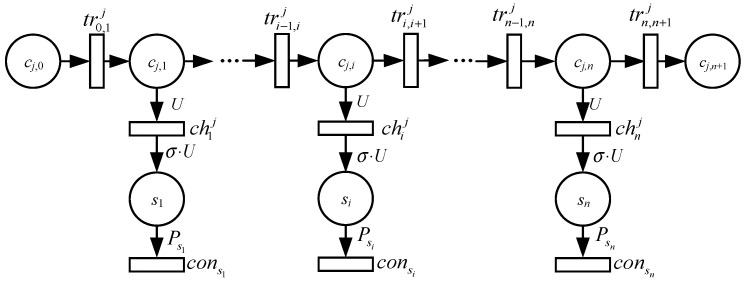
GSCCPNS model of wireless rechargeable sensor network.

The GSCCPNS model of the WRSN is illustrated in Figure 2. The place $c_{j,i}$ represents the energy of MV $c_{j}$ when located at node $s_{i}$. The places $c_{j,0}$ and $c_{j,n+1}$ represent the energy of MV $c_{j}$ when leaving and returning to the base station, respectively. The place $s_{i}$ represents the energy of the *i*th node. Transition $tr_{i-1,i}^{j}$ represents the movement of the MV $c_{j}$ from node $s_{i-1}$ to node $s_{i}$. Transition $ch_{i}^{j}$ represents the charging of node $s_{i}$ by MV $c_{j}$, while $con_{s_{i}}$ represents the energy consumption transition of node $s_{i}$.

Arc weight $W\left(tr_{i-1,i}^{j},c_{j,i}\right)=-\delta$ indicates the energy consumed per second by the MV during movement. $W\left(c_{j,i},ch_{i}^{j}\right)=U$ is the charging power of the MV, $W\left(ch_{i}^{j},s_{i}\right)=\sigma \cdot U$ is the power received by the node, σ is the reception efficiency, and $W\left(s_{i},con_{s_{i}}\right)=P_{s_{i}}$ is the power consumption of the sensor node.

Let $d_{i-1,i}^{j}$ represent the distance that the MV travels between adjacent nodes on its charging path. The time for the MV $c_{j}$ to move from node $s_{i}$ to node $s_{i+1}$ is given by:

(3) $$\tau_{i,i+1}^{j}=d_{i,i+1}^{j}/v$$

Thus, the total travelling time for the MV $c_{j}$ in one time slot is:

(4) $$\tau_{mov}^{j}=\sum_{i=0}^{n}\frac{d_{i,i+1}^{j}}{v}$$

The energy consumed during travelling is:

(5) $$E_{mov}^{j}=\delta \cdot \tau_{mov}^{j}$$

Due to the inherent characteristics of lithium batteries, the charging speed significantly decreases when the voltage reaches around 90%, and partial charging can extend the battery life [3]. Therefore, in this study, the nodes were partially charged. Let $\tau_{i}^{j}$ denote the charging time of MV $c_{j}$ at sensor node $s_{i}$. The total charging duration for nodes in one time slot is $\tau_{char}^{j}$. The energy consumed by $c_{j}$ for charging can be calculated as follows:

(6) $$E_{char}^{j}=U\cdot \tau_{char}^{j}=\sum_{i=1}^{n}U\tau_{i}^{j}$$

The time at which MV $c_{j}$ arrives at node $s_{i}$ is $t_{i}^{j}$. At this time, the energy of *c_j_* is represented by $M(c_{j,i}\_t_{i}^{j})$. After a charging duration of $\tau_{i}^{j}$, the energy of $c_{j}$ can be represented by $M\left[c_{j,i}\_(t_{i}^{j}+\tau_{i}^{j})\right]$.

The arc weight between the place of the MV and the transition of movement can be represented as follows:

(7) $$W\left(c_{j,i},tr_{i,i+1}^{j}\right)=-M\left[c_{j,i}\_(t_{i}^{j}+\tau_{i}^{j})\right]/\tau_{i,i+1}^{j}$$

Equation (7) indicates that after the MV $c_{j}$ reaches node $s_{i+1}$, the marking of place $c_{j,i}$ is zero.

If the MV $c_{j}$ stays at the base station for a duration of $\tau_{vac}^{j}$, then the total charging duration for *c_j_* in one time slot can be expressed as follows:

(8) $$T_{0}=\tau_{vac}^{j}+\tau_{mov}^{j}+\tau_{char}^{j}$$

where $\tau_{mov}^{j}=\sum_{i=0}^{n+1}\tau_{i,i+1}^{j}$, $\tau_{char}^{j}=\sum_{i=1}^{n}\tau_{i}^{j}$.The main components of GSCCPNS are described in detail in Table 2.

Given the scale of the wireless sensor network, node locations, energy consumption rates, base station location, and the energy capacity of MVs, this study aimed to address the problem of obtaining an optimal mobile charging scheme. Due to the limited energy of MVs, uneven distribution of sensor nodes, and significant differences in energy levels, the following factors need to be considered in charging scheduling:

- how to determine the number of MVs to deploy based on the number of nodes and their energy consumption;
- how to select nodes for charging to ensure no node dies, minimize the movement distance of MVs, and improve their charging benefits;
- how to allocate charging tasks to reduce the waiting time for subsequent nodes.

To address these issues, this paper proposes a charging scheduling strategy based on the energy consumption transition firing time. The main idea is to first group nodes according to their remaining lifetimes, placing nodes with similar lifetimes in the same group. Then, the number of MVs required is calculated based on the total energy consumption. Finally, intelligent algorithms are used to optimize the sequence of mobile transitions and the timing of charging transitions.

## 4. Problem Statement

This section first determines the scale of nodes that need charging in each time slot based on the firing time of consumption transition for the nodes and accordingly determines the number of MVs required. Next, the Mobile Charging Transition Firing Sequence Optimization Problem is proposed.

### 4.1. Node Place Selection

The remaining energy of nodes is often a crucial factor in charging planning, but the energy consumption of nodes varies depending on the amount of information they process. Consequently, nodes with low remaining energy might not necessarily deplete their energy soon, and nodes with high remaining energy might not necessarily operate for a long time. Therefore, using node lifetime as a basis for charging scheduling is more reasonable. In the GSCCPNS model, the remaining lifetime of a node is represented by the firing time of consumption transition (FTCT). Thus, this paper takes FTCT as an important basis for optimizing the mobile charging transition sequence and proposes a node place selection strategy.

Since node places represent the energy of sensors, the firing duration of the energy consumption transition $con_{s_{i}}$, which indicates the node’s lifespan, can be derived based on the arc weight $W\left(s_{i},con_{s_{i}}\right)$ when the charging transition $ch_{i}^{j}$ is not enabled. The integer ratio of the maximum FTCT to the minimum FTCT is used as the basis for grouping node places. Assume $l_{\min}$ and $l_{\max}$ are the minimum and maximum FTCT of node places when fully charged. To ensure that no node dies, the interval between any node’s two charging times should not exceed its FTCT. Let us define one time slot $T_{0}$ as $l_{\min}$. The ratio *K* is given by $K=\left\lfloor l_{\max}/l_{\min}\right\rfloor$, and it satisfies the following condition:

(9) $$kl_{\min}\leq l_{s_{i}}<\left(k+1\right)l_{\min},\;k\in K$$

In this context, $l_{s_{i}}$ represents the firing time of the energy consumption transition for place $s_{i}$ with unable charging transition. Nodes whose places satisfy the condition in Equation (9) can be grouped into node place groups $G_{k}$, with a maximum of *K* such groups. To ensure that the nodes in group $G_{k}$ receive energy replenishment, they must be charged once every *k* time slots. This means the energy replenishment period is $kl_{\min}$. Thus, any node at the beginning of its time slot will have an FTCT greater than $l_{\min}$ and less than or equal to $2l_{\min}$. Consequently, even if a node is charged last within a time slot, it will not die due to energy depletion.

### 4.2. Number of MV Places

From the above analysis, it can be concluded that the lifetime of a sensor node is not affected by the timing of its charging within its designated time slot. The earlier a node is charged, the less energy it needs to replenish; conversely, the later it is charged, the more energy it needs. Assuming the node that is charged the latest in group $G_{k}$ is located very close to the base station, the distance between them is $d_{\min}$. Thus, the interval between two charging times for this node is $kl_{\min}-d_{\min}/v$. The maximum energy demand for this node can be calculated as follows:

(10) $$E_{\max}=\underset{k\in K}{\max}\left\{\left(kl_{\min}-d_{\min}/v\right)P_{k}\right\}$$

where $P_{k}$ is the power consumption rate of the node. On the other hand, within group $G_{k}$, there must be a node that is charged first. This node, requiring the least energy replenishment, is also the closest to the base station, with a distance of ${d^{\prime }}_{\min}$ between them. Assuming the MV leaves the base station at time $t_{0}$, and the power consumption of this node is ${P^{\prime }}_{k}$, the minimum energy demand for this node can be calculated as follows:

(11) $$E_{\min}=\underset{k\in K}{\min}\left\{\left(\left(k-1\right)l_{\min}+t_{0}+{d^{\prime }}_{\min}/v\right){P^{\prime }}_{k}\right\}$$

where $\left(k-1\right)l_{\min}$ represents the time interval from the end of the last charging slot to the beginning of the current charging slot, during which the node only consumes energy without being charged.

Equations (10) and (11), respectively, provide the maximum and minimum energy demands of the node places. Based on this, the range for the number of MVs required can be determined.

(12) $$\frac{n\left(\delta \cdot \bar{d}/v+E_{\min}/\sigma \right)}{k_{H}\left(c_{j}\right)-\delta \cdot \bar{d}/v}\leq m\leq \frac{n\left(\delta \cdot \bar{d}/v+E_{\max}/\sigma \right)}{k_{H}\left(c_{j}\right)-\delta \cdot \bar{d}/v}$$

where $\bar{d}$ is the average distance the MV moves between any two nodes, $\delta \cdot \bar{d}/v$ is the average energy consumption for movement, and $k_{H}\left(c_{j}\right)$ is the upper bound of the place capacity $c_{j}$, representing the battery capacity of MV. $n\left(\delta \cdot \bar{d}/v+E_{\min}\right)$ and $n\left(\delta \cdot \bar{d}/v+E_{\max}\right)$ are the minimum and maximum energy demands of all nodes, respectively. $k_{H}\left(c_{j}\right)-\delta \cdot \bar{d}/v$ represents the upper limit of the energy available for charging via a single MV.

### 4.3. Problem Definition

This paper measures the charging benefit of MVs using the energy utilization rate ϕ. The charging optimization objective is defined as maximizing charging benefit to achieve the optimal usage of MVs. The problem definition is provided as follows.

**Definition** **4.** *The Firing Sequence Optimization of Mobile Charging Transition (FSOMCT) problem involves finding the optimal sequence of MV transitions and the timing of charging transitions within the constraints of charging time slots, node energy consumption transition durations, and the capacities of MVs and sensor nodes. The goal is to maximize charging benefit. The formal specification of the FSOMCT is as follows:*

(13) $$\max\phi =\frac{\sum_{i=1}^{N_{j}}\sum_{j=1}^{M}W\left(c_{j,i},ch_{i}^{j}\right)\cdot \tau_{i}^{j}}{\sum_{j=1}^{M}\left[M_{0}\left(c_{j,0}\right)-M\left(c_{j,n+1}\_t_{n+1}^{j}\right)\right]}$$

subject to

(14) $$M\left(c_{j,n+1}\_t_{n+1}^{j}\right)\geq 0,j=1,2,\cdots ,M$$

(15) $$T_{0}=\tau_{vac}^{j}+\tau_{mov}^{j}+\tau_{char}^{j}$$

(16) $$k\cdot l_{\min}\leq M\left[s_{i}\_\left(t_{i}^{j}+\tau_{i}^{j}\right)\right]/P_{s_{i}}\leq \left(k+1\right)\cdot l_{\min}$$

(17) $$\sum_{i=0}^{N_{j}+1}\sum_{j=1}^{M}x_{gij}=1,\forall g$$

(18) $$\sum_{i=0}^{N_{j}+1}x_{0ij}=1\;and\;\sum_{g=0}^{N_{j}+1}x_{g0j}=1,\forall j$$

(19) $$\sum_{i=0}^{N_{j}+1}x_{gij}-\sum_{g=0}^{N_{j}+1}x_{igj}=0,\forall g,j$$

In Equation (13), $M_{0}\left(c_{j,0}\right)-M\left(c_{j,n+1}\_t_{n+1}^{j}\right)$ refers to the energy consumed by MV $c_{j}$ within one time slot, and $M_{0}\left(c_{j,0}\right)$ is the energy at the time of departure, when it is generally considered to be fully charged. $N_{j}$ indicates the number of sensors that MV $c_{j}$ needs to serve. Equation (14) shows that the MV has to return to the base station with enough energy. Equation (15) defines the total duration of a time slot, and Equation (16) determines the range of energy charging for a node. In Equation (17), $x_{gij}$ is a binary decision variable. If there is a movement transition $tr_{g,i}^{j}$ between places $s_{g}$ and $s_{i}$, meaning MV $c_{j}$ moves to node $s_{i}$ after charging node $s_{g}$, then $x_{gij}=1$; otherwise, $x_{gij}=0$. Equation (17) is the single-visit constraint for nodes, Equation (18) implies that the MV departs from and eventually returns to the base station, and Equation (19) ensures that the route of each MV is logically continuous.

**Theorem** **1.** *The FSOMCT problem is NP-hard.*

**Proof.**  The MV places can be considered analogous to cargo vehicles, and the sensor node places can be viewed as customers needing service. The sequence of mobile charging transition firings can be seen as the order of customer visits. □

Specifically, in the FSOMCT, the charging capacity of each MV corresponds to the vehicle capacity in the Capacitated Multi-Depot Vehicle Routing Problem (CMDVRP), the service time or energy consumption required by each sensor node is equivalent to the customer demand in the CMDVRP, and the vehicle’s charging path corresponds to the vehicle routing problem in the CMDVRP. Additionally, the multi-vehicle and the depot in the CMDVRP are similar to the setup of multiple MVs and the base station in the FSOMCT.

Therefore, by mapping the FSOMCT to the CMDVRP, it can be seen that these two problems share the same structural characteristics in terms of computational complexity. Since the CMDVRP has been proven to be NP-hard, and the FSOMCT can be reduced to the CMDVRP in polynomial time, the FSOMCT is also an NP-hard problem.

## 5. Proposed Algorithm

Based on the previous section’s description, it is clear that the problem addressed in this paper is to find the optimal sequence of mobile charging transitions, which is also NP-hard. This involves determining the traversal order of the MV to the nodes and the charging times. Intelligent optimization algorithms have proven effective in solving such problems. Among them, ant colony optimization (ACO) is well-suited for finding the shortest path, while the fireworks algorithm (FWA) offers strong global search capabilities. Therefore, this paper proposes a hybrid algorithm combining ACO and FWA, leveraging the strengths of both. The ant colony algorithm is used to effectively explore paths and enhance convergence speed through pheromone reinforcement, while the fireworks algorithm is employed to increase solution diversity and improve global optimization capabilities.

### 5.1. Improvement of Ant Colony Optimization

Ant colony optimization (ACO) is inspired by the foraging behavior of ants in nature, where they find the shortest path from the food source to their nest. Ants are able to find this shortest path without any external clues by laying down pheromones to communicate with each other. Thus, even if the environment changes, they can still find the shortest path. The concentration of pheromones on a path determines the probability of ants choosing that path [25]. Therefore, there are two crucial processes in ACO. One of these is determining the next node for the ant to move to, and the other is updating the pheromone trail. At time *t*, ant *w* is at node *g* and moves to the next node *i* according to Equation (20):

(20) $$P_{gi}^{w}\left(t\right)=\{\begin{array}{cc} \frac{\tau_{gi}^{\alpha }\left(t\right)\cdot \eta_{gi}^{\beta }\left(t\right)}{\sum_{i\subset N_{i}^{w}}\tau_{gi}^{\alpha }\left(t\right)\cdot \eta_{gi}^{\beta }\left(t\right)},\; & i\subset allowed_{w}\\ 0, & otherwise \end{array}$$

where $\tau_{gi}\left(t\right)$ represents the pheromone intensity on the edge (g,i), $\eta_{gi}$ is the inverse of the distance between nodes g and i, α is the relative importance of the pheromone trail, and β is the relative importance of visibility. $N_{i}^{w}$ represents the set of nodes yet to be visited, and $allowed_{w}$ denotes the set of nodes that ant w can choose from.

The concentration of pheromones is inversely proportional to the path length. The more pheromones present, the shorter the path, as more ants will choose this path. Conversely, fewer pheromones indicate a longer path, with fewer ants selecting it. To prevent excessive pheromone accumulation, which can reduce the influence of heuristic factors, the pheromone levels need to be updated according to the following formula:

(21) $$\tau_{gi}\left(t+1\right)=\left(1-\rho \right)\tau_{gi}\left(t\right)+\sum_{w=1}^{m}\Delta \tau_{gi}^{w}\left(t\right)$$

(22) $$\Delta \tau_{gi}^{w}\left(t\right)=\{\begin{array}{c} \frac{Q}{L_{w}},\;if\;ant\;w\;transfer\;the\;edge\;\left(g,i\right)\\ 0,\;otherwise \end{array}$$

where ρ is the parameter controlling the pheromone evaporation rate; $\Delta \tau_{gi}^{w}\left(t\right)$ represents the amount of pheromone created by ant w on edge (*g*,*i*); Q is a constant related to the amount of pheromone deposited by the ant; $L_{w}$ is the length of the path traversed by ant w in the current cycle.

However, basic ACO is prone to falling into local optima and has a long global convergence time during the search process, making it less suitable for practical applications. To address this, ACO is improved in two ways. Firstly, an elite strategy is introduced, adding extra pheromones to the optimal path $L_{opt}$. The improved fireworks algorithm (described in Section 5.2) is used to optimize the current path, obtaining the local optimal solution $L_{local}$ ($L_{local}=L_{opt}$). To make $L_{opt}$ more attractive to the ants in the next cycle, the local pheromone update formula can be expressed as follows:

(23) $$\tau_{gi}\left(t+1\right)=\rho \tau_{gi}\left(t\right)+\sum_{w=1}^{m}\Delta \tau_{gi}^{w}\left(t\right)+\left(1-\rho \right)\tau_{gi}^{*}\left(t\right)$$

(24) $$\tau_{gi}^{w}\left(t\right)=\frac{Q}{L_{local}}$$

(25) $$\Delta \tau_{gi}^{w}\left(t\right)=\{\begin{array}{c} \frac{Q}{L_{w}},\;ant\;w\;traverses\;edge\left(g,i\right)\\ 0,\;otherwise \end{array}$$

(26) $$\tau_{gi}^{*}\left(t\right)=\sigma \cdot \frac{Q}{L_{local}}$$

where ρ, $\tau_{gi}\left(t\right)$, $\Delta \tau_{gi}^{w}\left(t\right)$, and Q have the same meanings as in Equations (20)–(22). $\Delta \tau_{gi}^{*}\left(t\right)$ represents the additional amount of pheromone introduced by elite ants, and σ is the number of elite ants.

Secondly, the Max–Min Ant System (MMAS) is introduced to limit the range of pheromone concentrations. However, introducing the elite ant strategy into the local pheromone update formula may lead to excessive growth of pheromone on certain paths. Therefore, the pheromone values are restricted to the interval $\left[\tau_{min},\tau_{max}\right]$ to prevent the algorithm from stagnating. The global update formula can be expressed as follows:

(27) $$\tau_{gi}\left(t+1\right)=\rho \tau_{gi}\left(t\right)+\Delta \tau_{gi}^{global}\left(t\right)$$

(28) $$\Delta \tau_{gi}^{global}\left(t\right)=\frac{Q}{L_{global}}$$

where $L_{global}$ is the global optimal path. The pheromone values are restricted to the interval $\left[\tau_{min},\tau_{max}\right]$. After one cycle, if $\tau_{gi}\geq \tau_{max}$, then $\tau_{gi}=\tau_{max}$; if $\tau_{gi}\leq \tau_{min}$, then $\tau_{gi}=\tau_{min}$.

### 5.2. Improvement of the Fireworks Algorithm

The fireworks algorithm (FWA) is a novel intelligent algorithm proposed by Tan and Zhu in 2010 [26]. In FWA, fireworks are considered as feasible solutions to the optimization problem, and the process of searching the neighborhood is viewed similarly to the process of generating sparks from fireworks.

To adapt the FWA to the proposed optimization problem, this paper improves the basic FWA. The algorithm steps are as follows.

First, initialize the fireworks population and parameters. Second, calculate the number of explosion sparks $F_{g}$ and the explosion amplitude $A_{g}$ for each firework. Then, select fireworks with smaller explosions to perform the explosion operation and generate sparks. Third, use the mutation operation to generate sparks $M_{g}$, where $M_{g}=\alpha \cdot F_{g}$, and α is a real number in the interval [0,1]. Finally, according to the selection strategy, select the best fireworks or sparks as the next generation of fireworks, and repeat the process until the stopping criteria are met. The framework of the improved fireworks algorithm is shown in Algorithm 1.
**Algorithm 1** Improved fireworks algorithm**Input:** Population size *N*, number of iterations *T*, number of sparks *F*, explosion amplitude *A* **Output:** Local optimal solution 1:  Initialization 2:  **for** *t* = 1 to *T* 3:    **for** *g* = 1 to *F_g_* **do** 4:      generate a spark by the explosion operator 5:    **end for** 6:    **for** *k* = 1 to *M_g_* **do** 7:      generate a spark by the mutation operator 8:    **end for** 9:    select *N* fireworks and sparks as the child generation 10:  **end for**

During each iteration of Algorithm 1, the process is divided into three stages as follows:

Initialization: Define the parameters of the fireworks algorithm and determine the first generation of fireworks.

Local Search: Perform a local search through explosion and mutation operations. Before the search procedure, determine the search range and the number of searches. For minimization problems, the number of explosion sparks $F_{g}$ and the explosion amplitude $A_{g}$ of a firework are calculated as follows:

(29) $$F_{g}=M\times \frac{y_{\max}-f\left(x_{g}\right)+\varepsilon }{\sum_{g=1}^{N}\left(y_{\max}-f\left(x_{g}\right)\right)+\varepsilon }$$

(30) $$A_{g}=\hat{A}\times \frac{f\left(x_{g}\right)-y_{\min}+\varepsilon }{\sum_{g=1}^{N}\left(f\left(x_{g}\right)-y_{\min}\right)+\varepsilon }$$

where M is the total number of explosion sparks used to adjust the number of sparks generated by the explosion, and $\hat{A}$ is the maximum radius used to adjust the size of the explosion radius. $f\left(x_{g}\right)$ is the objective function fitness value of firework $x_{g}$. $y_{\max}=\max\left(f\left(x_{g}\right)\right)$, $y_{\min}=\min\left(f\left(x_{g}\right)\right)$ and ε is a very small number used to prevent the denominator from being zero.

To prevent fireworks with good fitness from generating too many explosion sparks and fireworks with poor fitness from generating too few explosion sparks, upper and lower limits are set for the number of explosion sparks. Parameters a and b (0<a<b<1) are used, and the number of sparks generated by firework $x_{g}$ is calculated using Equation (31):

(31) $${\hat{F}}_{g}=\{\begin{array}{c} round\left(a*M\right),\;F_{g}<aM\\ round\left(b*M\right),F_{g}>bM,\;a<b<1\\ round\left(F_{g}\right),\;F_{g}>aM \end{array}$$

To ensure that the solutions randomly sampled from the solution space are locally optimal, the 2-opt algorithm is introduced. This is a local search algorithm that involves two types of exchanges: intra-path 2-opt exchange and inter-path 2-opt exchange. In this paper, the former is introduced.

After the explosion operation, the mutation operation generates Gaussian sparks to increase the diversity of solutions. The swap operator is then introduced into the mutation operation. The swap operator can be described as follows. Given a set of feasible solutions represented as $\pi =\left\{\pi_{1},\pi_{2},\cdots ,\pi_{n}\right\}$, the swap operator randomly selects two nodes $\pi_{g}$ and $\pi_{i}$ (g<i) and swaps their positions to obtain a new set of solutions $\pi^{\prime }=\left\{\pi_{1},\cdots ,\pi_{g-1},\pi_{i},\pi_{g+1},\cdots ,\pi_{i-1},\pi_{g},\pi_{i+1},\cdots ,\pi_{n}\right\}$.

Selection: After the series of operations described above, the fireworks themselves, along with the explosion sparks and Gaussian mutation sparks, collectively form the candidate set of solutions. To pass on excellent individuals from the fireworks population to the next generation, a certain number of individuals need to be selected from the candidate set as the next generation of fireworks. Assume W is the candidate set and N is the number of fireworks. The best solution $x_{b}$ ($x_{b}\in W$) and N−1 other solutions are selected as the next generation of fireworks. The remaining N−1 fireworks are selected using the roulette wheel method. The selection probability of a solution $x_{b}$ is given as follows:

(32) $$P\left(x_{g}\right)=\frac{R\left(x_{g}\right)}{\sum_{x_{i}\in W}R\left(x_{i}\right)}$$

(33) $$R\left(x_{g}\right)=\sum_{x_{i}\in K}d\left(x_{g}-x_{i}\right)=\sum_{x_{i}\in K}\left\|x_{g}-x_{i}\right\|$$

where $R\left(x_{g}\right)$ represents the distance between the current individual W and all other individuals except $x_{g}$.

### 5.3. Structure of MTS-HACO

In this section, a new algorithm named MTS-HACO (Mobile Transition Sequence Hybrid Ant Colony Optimization) is presented. The main objective of this method is to provide a novel hybrid approach to solve the FSOMCT problem. The algorithmic framework of MTS-HACO is shown in Algorithm 2.
**Algorithm 2** MTS-HACO algorithm**Input:** num_ants, num_nodes, initial_pheromone, max_iterations, constraints, stop_condition **Output:** mobile transition sequence, firing time of charging transition 1:  **while** (stop_condition_not_satisfied) **do** 2:     Initialization 3:     **for** each ant *w* **do** 4:        distribute_ant_randomly (*w*) 5:        include_location_in_tabu_table (*w*) 6:        calculate_state_transition_probability (*w*) 7:        **if** (constraint_satisfied) **then** 8:          place_node_in_tabu_table (*w*) 9:        **else** 10:          continue 11:       **end if** 12:        **if** (traversal_completed) **then** 13:          save_optimal_solution() 14:          update_local_pheromone() 15:        **end if** 16:     **end for** 17:     update_global_pheromone() 18:  **end while** 19:  output_optimal_mobile transition sequence and firing time of charging transition

As shown in Algorithm 2, after initializing the parameters, m ants are randomly placed on each node, and their positions are recorded in the corresponding tabu list. The next node i for ant w to visit is chosen based on the state transition probability given by Formula (20). Nodes that satisfy the constraints are added to $tabu_{w}$ until the paths for all ants are constructed. FWA is then used to locally optimize the paths constructed by each ant, obtaining the local optimal solution $L_{local}$. The elite ant strategy is applied to the local optimal paths, and the local pheromone is updated according to Formula (23). By calculating the total shortest path, the global optimal solution $L_{global}$ is obtained, and the global pheromone is updated according to Formula (27). This process continues until the termination condition $NC=NC_{\max}$ is met, at which point the loop stops and the optimal solution is output.

MTS-HACO improves traditional ACO by introducing the elite strategy and the Max-Min Ant System and uses the improved FWA to optimize the paths constructed by each ant. These strategies increase the computational load of each iteration. However, the overall complexity is comparable to that of basic ACO. The total time complexity of the ACO algorithm with the elite strategy and MMAS is still mainly influenced by the number of ants, the number of nodes, and the number of iterations, and can be expressed as $O\left(T\times \left(m\times n+n^{2}+N\times F+M\right)\right)$. Here, m×n represents the complexity of ants constructing solutions, $n^{2}$ represents the complexity of limiting pheromone range on all edges, N×F refers to the explosion operation for each firework multiplied by the number of fireworks, *M* indicates the mutation operations, and *T* represents the number of iterations. Therefore, the time complexity of the MTS-HACO algorithm can be simplified to *O*(*n*^3^).

Compared with traditional ACO or other metaheuristic algorithms such as genetic algorithms (GAs), MTS-HACO incorporates a multi-search mechanism that improves solution quality but increases computational cost. While classic ACO has a complexity of O(m×n), MTS-HACO’s additional local search steps introduce a slightly higher complexity. However, this increase is offset by the improved convergence speed and solution accuracy, particularly for large-scale problems like ours.

## 6. Performance Evaluation

This study simulated an environmental monitoring sensor network with N = [50, 100, 150, 200] randomly deployed nodes. The sensing area had a radius of 250 m, with a base station at the center. Each node had an initial energy of 100 joules, and the power consumption of the node ranged from 0.01 J/s to 0.03 J/s. Each MV had an energy capacity of 10 kJ, with a charging power of 10 W and a charging efficiency σ=0.6. All MVs moved at a speed of 8 m/s, and the energy consumption per MV was 16 J/s.

**Figure 3:**
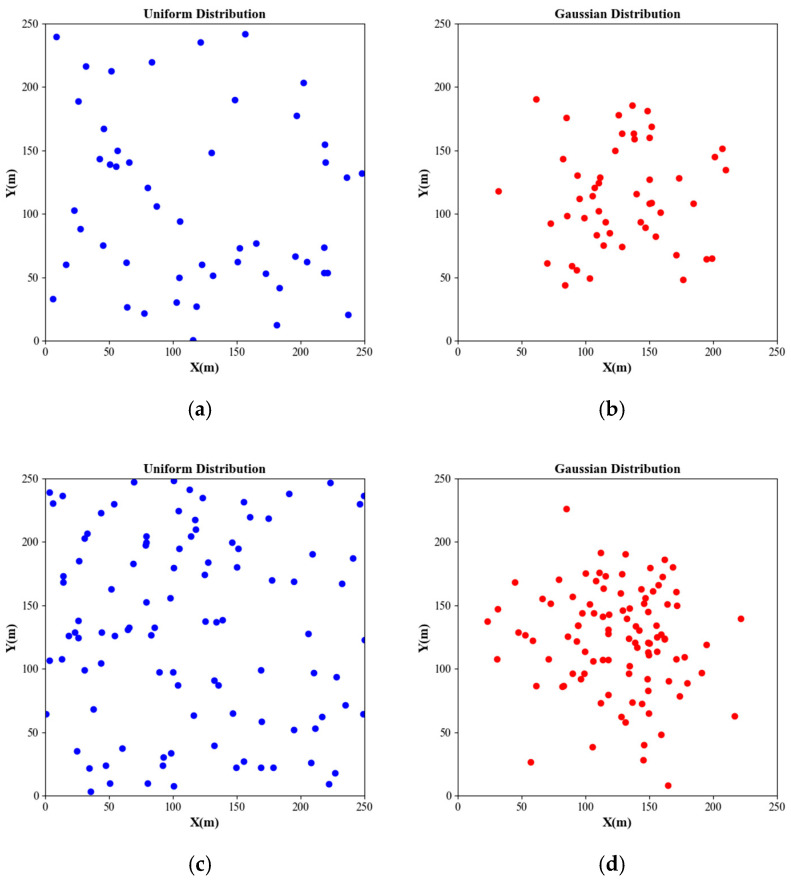
Network topologies with different nodes’ numbers and distribution. (**a**) Net1, N = 50; (**b**) Net2, N = 50; (**c**) Net1, N = 100; (**d**) Net2, N = 100.

To verify the adaptability of the proposed scheme to any node distribution, two types of networks were established in the simulation (referred to as Net1 and Net2). In Net1, the nodes were uniformly distributed, while in Net2, the nodes followed a Gaussian distribution. The network topologies for N = 50 and N = 100 are shown in Figure 3.

Three criteria were used to demonstrate the performance of the MTS-HACO algorithm, the PE-FWA algorithm, and the periodic algorithm.

- Average Travelling Distance of MV: This was the ratio of the total travelling distance of the MV during the charging path to the number of nodes it charged, represented as $D_{j}/N_{j}$, where $D_{j}$ is the travelling distance of the MV during a certain time slot, and $N_{j}$ is the number of nodes it charges. By calculating the average travelling distance, the performance of the MV was assessed. A shorter average distance indicated that the MV was able to efficiently charge nodes with relatively low energy consumption. Conversely, if the average distance was long, further optimization of the MV’s movement transition firing sequence would be needed to reduce the travelling distance for lower energy consumption.
- Number of Energy-Depleted Sensor Nodes: This referred to the number of sensor nodes in the network that had their energy depleted due to not being charged in time. The greater the number of energy-depleted sensor nodes, the worse was the network performance. Therefore, the number of dead nodes was an important metric for evaluating the MTS-HACO algorithm.
- Charging Benefit: This was the target of optimization in this study, representing the ratio of the energy consumed for charging to the total energy consumption. High charging benefit indicates effective utilization of the MVs for energy replenishment.

### 6.1. Charging Benfits

**Figure 4:**
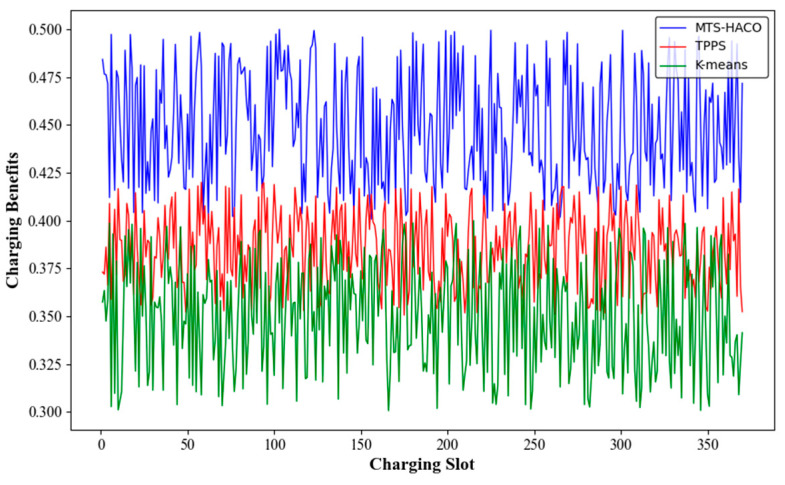
Charging benefits with Net1 in three recharging methods.

**Figure 5:**
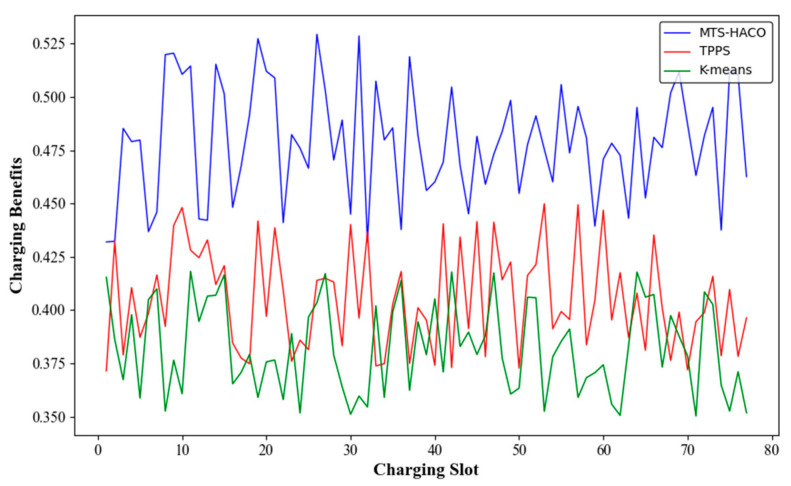
Charging benefits with Net2 in three recharging methods.

In this part of the simulation, the number of nodes was set to 100, and it was calculated that the complete charging cycles in Net1 and Net2 contained 370 and 77 time slots, respectively, requiring four MVs. After running the TPPS, K-Means, and MTS-HACO algorithms in Net1 and Net2, the charging benefit for each time slot was obtained, as shown in Figure 4 and Figure 5. TPPS and K-Means are both region-based charging task allocation methods [7,27]. K-Means divided the network into several subregions based on the node locations. Each MV was assigned to one of these regions and independently charged the nodes within that region, with the advantage of avoiding long-distance movement by a single MV. On the other hand, TPPS, in addition to network partitioning, introduced a group member adjustment strategy to minimize the energy consumption of the MVs.

The simulation results showed that, regardless of the network topology, the MTS-HACO algorithm had a significant advantage in terms of charging benefit. The average charging benefit of the MTS-HACO algorithm in Net2 was 6.7% higher than in Net1, due to the more concentrated distribution of nodes in Net2, which helped the MVs improve energy utilization. In Net1 and Net2, the average charging benefits of the MTS-HACO algorithm were 16.6% and 27.9% higher than TPPS, and 18.2% and 25.3% higher than K-Means, respectively. This improvement was because TPPS and K-Means did not consider the differences in energy demand between nodes when establishing MV trajectories; their optimization objectives were focused on balancing the energy consumption of MVs during movement.

**Figure 6:**
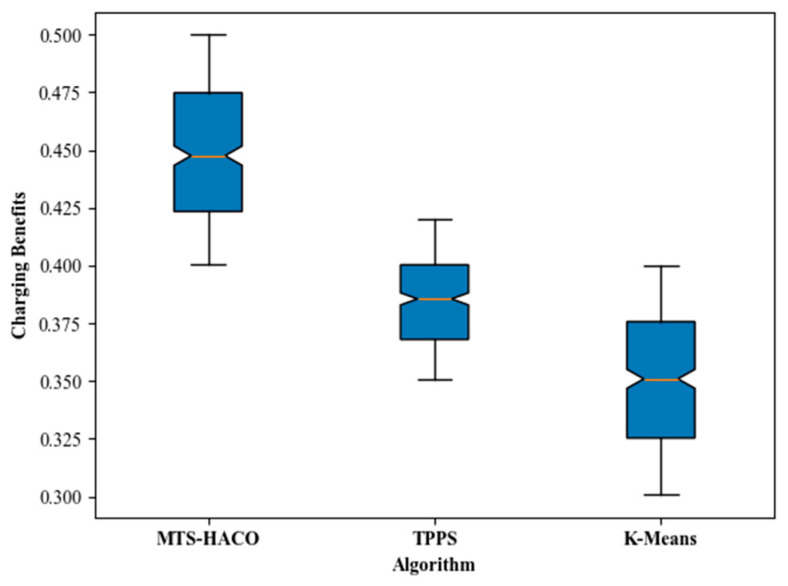
Box plot of charging benefits with Net1 in three recharging methods.

**Figure 7:**
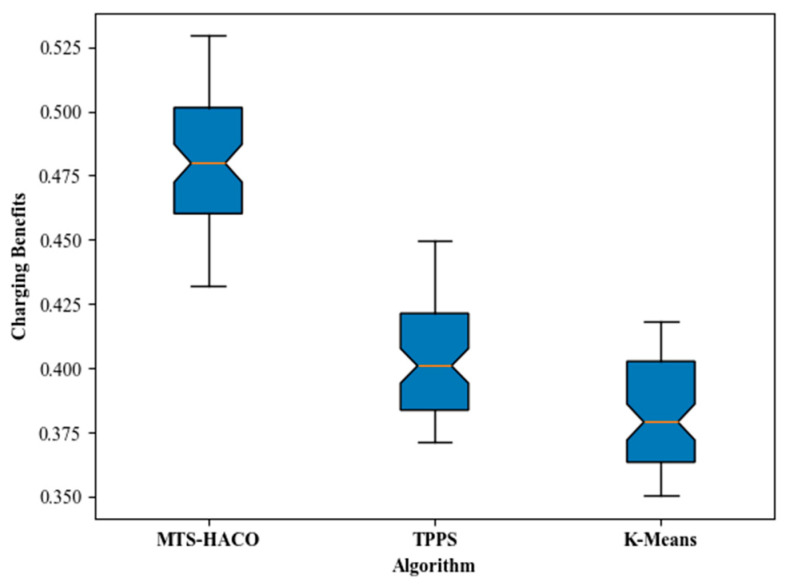
Box plot of charging benefits with Net2 in three recharging methods.

Additionally, as shown in Figure 6 and Figure 7, the optimal, median, and worst values of the HTS-HACO algorithm in both scenarios were significantly better than those of the other two algorithms.

### 6.2. Network Performance with Various Number of MVs

**Figure 8:**
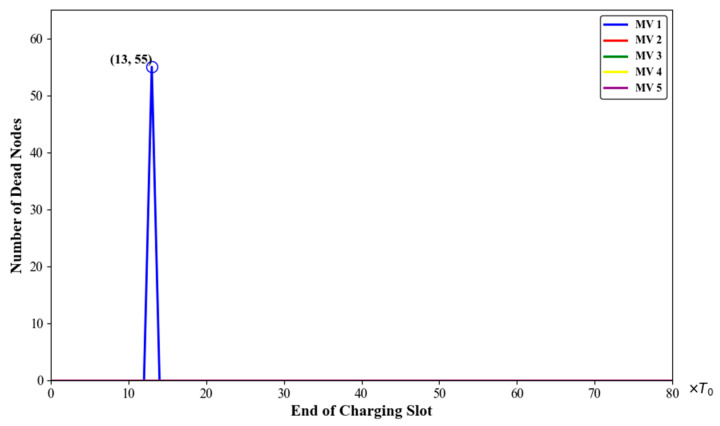
Number of dead nodes with different numbers of mobile vehicles.

**Figure 9:**
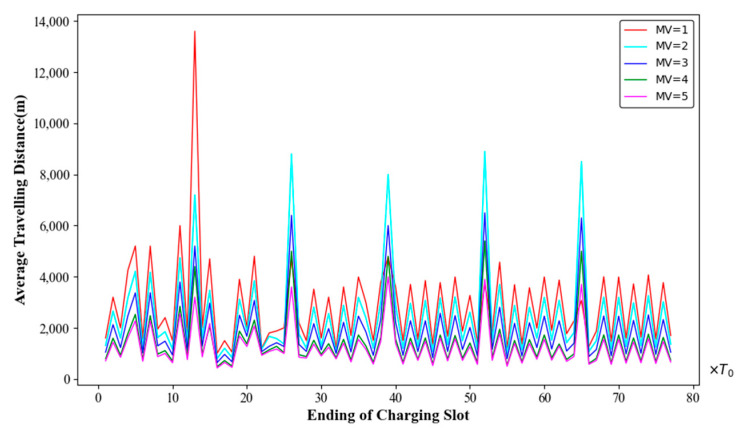
Average travelling distance with different numbers of mobile vehicles.

**Figure 10:**
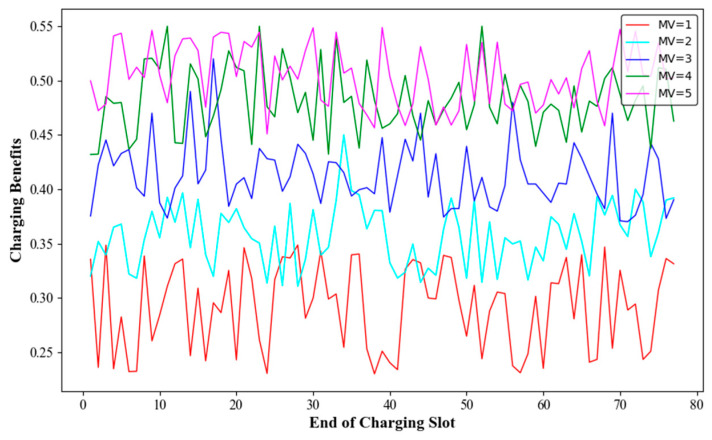
Charging benefits with different numbers of mobile vehicles.

In a network with 100 deployed nodes, it can be calculated using Equation (12) that the theoretically required number of MVs is three or four. Without loss of generality, we increased the number of MVs from one to five and recorded the average travelling distance of the MVs, the number of dead nodes, and the charging benefit of the MVs. The results are shown in Figure 8, Figure 9 and Figure 10.

As seen in Figure 8, when m=1, 72 nodes sent charging requests in the 13th time slot. At the same time, 55 nodes died due to not being charged in time. When m≥2, the node death rate in the network dropped to zero.

In Figure 9, when m=1, the average travelling distance of the MV was nearly the longest. However, the experimental results for the 26th, 39th, 52nd, and 65th charging time slots were inconsistent with the above conclusion. This discrepancy was due to the fact that many nodes died by the end of the 13th time slot. As a result, the number of charging nodes in the subsequent time slots decreased sharply, leading to a reduction in the MV’s average travelling distance. However, it is important to note that in other time slots, when m=1, the MV’s average travelling distance remained the longest.

When m≥2, the average travelling distance of the MVs decreased as m increased, indicating that increasing the number of MVs would be an effective way to enhance network performance. However, it should be noted that when m=3, the average travelling distance of the MVs was significantly shorter than when m=2, with an average reduction of 559 m. Conversely, when *m* = 5, the average travelling distance of the MVs was not significantly shorter than when m=4. This suggests that more MVs are not always better, and there is an optimal number of MVs for achieving the best performance.

Figure 10 shows the charging benefits of MVs with different numbers of MVs. When the numbers of MVs were one, two, three, four, and five, the average charging benefits were 29.2%, 35.8%, 41.3%, 48.1%, and 50.4%, respectively. When m=2, the charging benefit was relatively high, but the average travelling distance of the MVs was also longer. For 3≤m≤5, it was observed that when m=4, the charging benefit was the second highest, and the average travelling distance was close to that when m=5. Therefore, when deploying 100 nodes, setting the number of MVs to four would be more reasonable.

**Figure 11:**
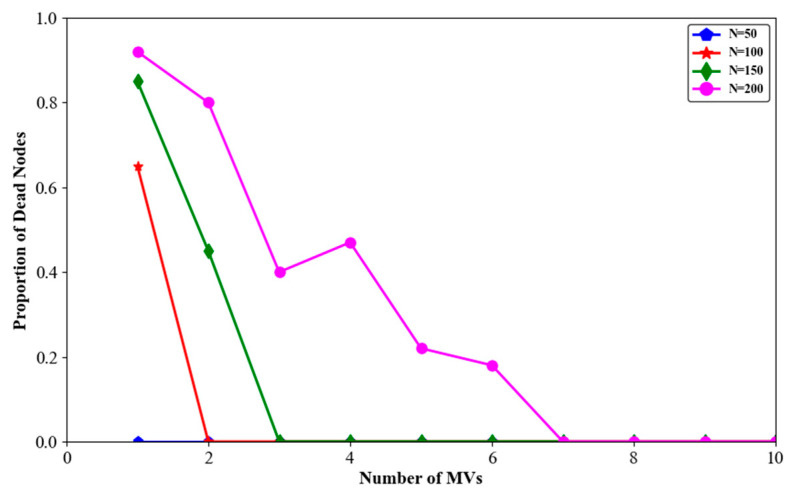
Proportion of dead nodes with different numbers of sensor nodes.

**Figure 12:**
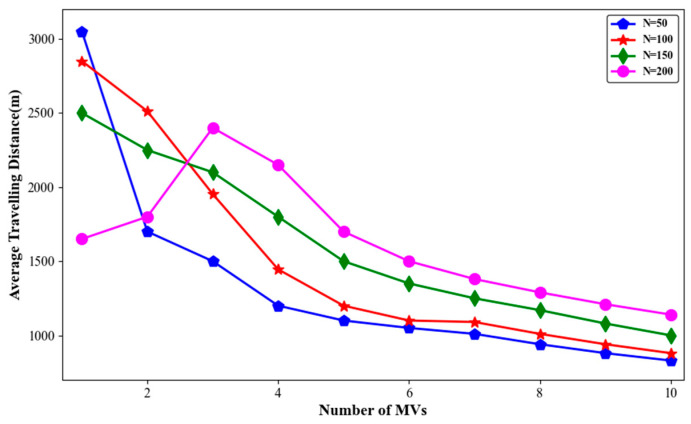
Average travelling distance with different numbers of sensor nodes.

**Figure 13:**
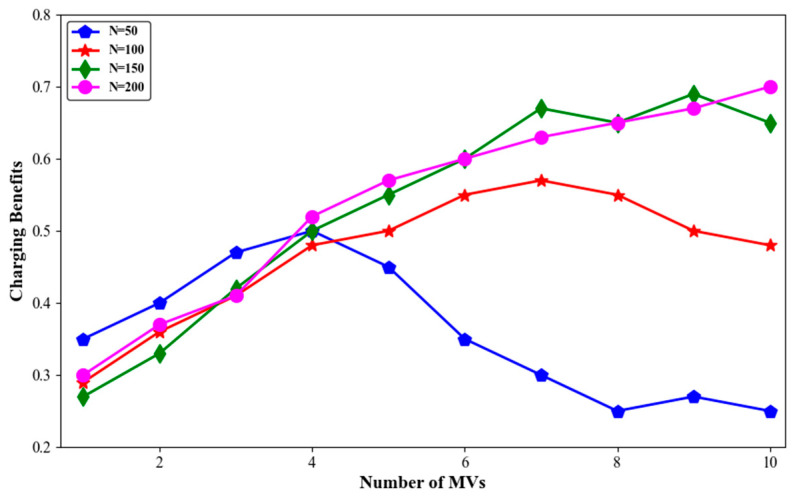
Charging benefits with different numbers of sensor nodes.

The simulation results for node death rate, average travelling distance of MVs, and average charging benefit with different numbers of nodes are shown in Figure 11, Figure 12 and Figure 13.

As shown in Figure 11, when the number of MVs remained constant, the more nodes in the network, the higher the node death rate. This was clearly due to the limited number and energy capacity of the MVs, which were unable to meet the increasing charging demands of the nodes. On the other hand, as the number of MVs increased, the node death rate in the network gradually decreased, regardless of the number of nodes. For example, in a network with N = 200 nodes, there were no node deaths when the number of MVs was seven.

As shown in Figure 12, when the number of MVs remained constant, the average travelling distance of the MVs generally increased with the number of nodes. It is noteworthy that when *m* = 1, the average travelling distance of MVs in a network with N = 50 was actually the highest. This was because, in networks with N = 100, 150, or 200 nodes, the node death rate was relatively high. As shown in Figure 9, when N = 200 and *m* = 1, more than 90% of the nodes in the network died. In such a scenario, the MV only needed to charge a few surviving nodes, which shortened the average travelling distance.

As seen in Figure 13, in a network with N = 50, the average charging benefit of MVs significantly improved when the number of MVs increased from one to four. As the number of nodes increased, correspondingly increasing the number of MVs significantly enhanced the average charging benefit.

### 6.3. Network Performance with Different Charging Strategies

In this section, the proposed MTS-HACO is compared with the PE-FWA and periodic methods [7,8]. In PE-FWA, nodes are clustered based on their locations, and when a cluster head sends a charging request, the MV charges all the nodes within that cluster. The clustering and grouping depend on both the request threshold of the cluster head and a unified charging threshold. Therefore, improper settings of these two thresholds may lead to excessive charging of nodes. In this method, the MV often needs to move back and forth between different cluster heads, resulting in a higher proportion of energy consumption due to movement.

These three strategies were all run in the same network. The charging times, energy changes, movement trajectories, and energy consumption of each MV were recorded during one cycle *T* (T=22,170s, $T=15\times T_{0}$, *T*_0_ = 1478s).

**Figure 14:**
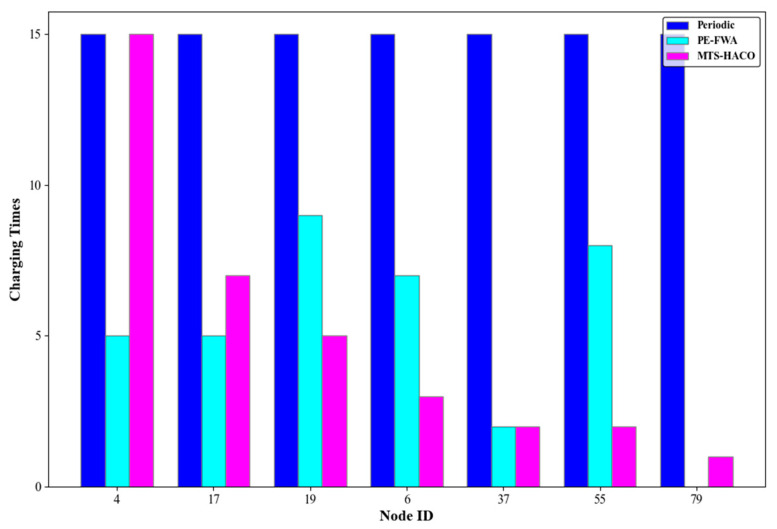
Charging times of the three algorithms.

**Figure 15:**
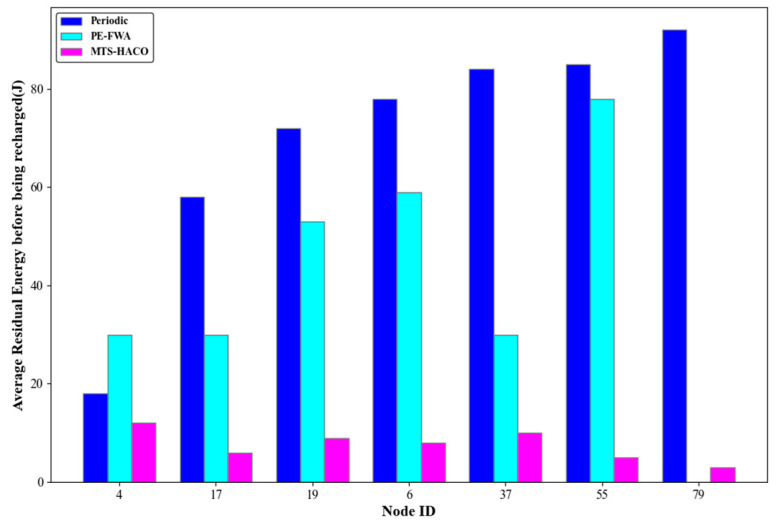
Average residual energy before being recharged for the three algorithms.

**Figure 16:**
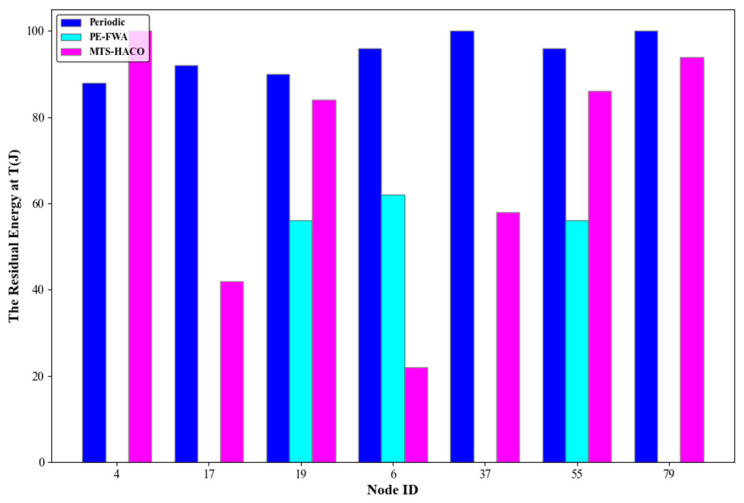
The residual energy at T for the three algorithms.

**Figure 17:**
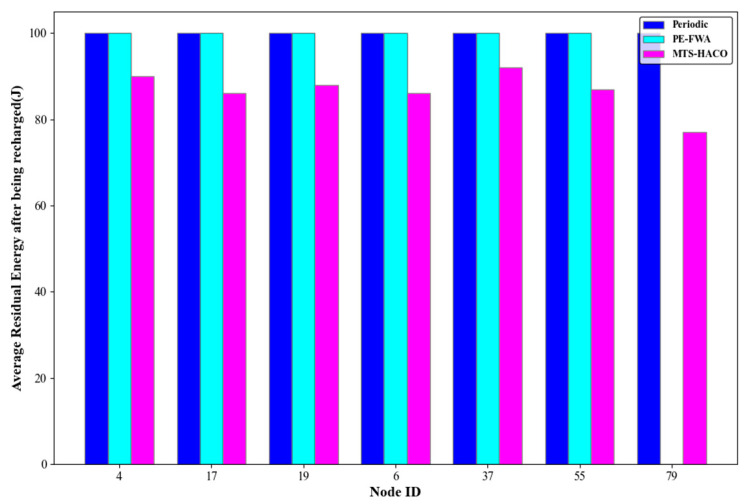
Average residual energy after being recharged for the three algorithms.

Assume that the network has 100 nodes, divided into seven non-empty node groups with sequence numbers 2, 3, 5, 7, 8, 9, and 14. One node was randomly selected from each group, with node IDs 4, 17, 19, 6, 37, 55, and 79, respectively. The number of charging times, the average remaining energy of each node before and after charging during cycle T, and the remaining energy of each node at the end of the cycle are shown in Figure 14, Figure 15, Figure 16 and Figure 17.

As shown in Figure 14, Figure 15 and Figure 16, all seven nodes under the MTS-HACO algorithm survived within a cycle, with fewer charging instances, and the average remaining energy before charging was less than 16% of their initial energy. The MTS-HACO algorithm performed better overall. In contrast, the periodic charging strategy involved the most charging instances among the three strategies, as it frequently replenished energy to ensure node survival, resulting in the nodes having the most remaining energy before charging. Additionally, under the PE-FWA strategy, nodes 4, 17, 37, and 79 had fewer charging instances than under MTS-HACO. However, these nodes died before the end of the cycle. Although nodes 6, 19, and 55 remained active throughout the cycle, they had more charging instances than under MTS-HACO. This was because, in the PE-FWA strategy, nodes were clustered based on their location, and when the cluster head sent a charging request, the MV charged all nodes within the cluster. As a result, nodes that did not send a charging request also received a charge.

Figure 17 shows the remaining energy of each node after charging. As can be seen from the previous figures, MTS-HACO minimized energy replenishment while ensuring that no nodes died, thereby significantly reducing the waiting time for subsequent nodes.

**Figure 18:**
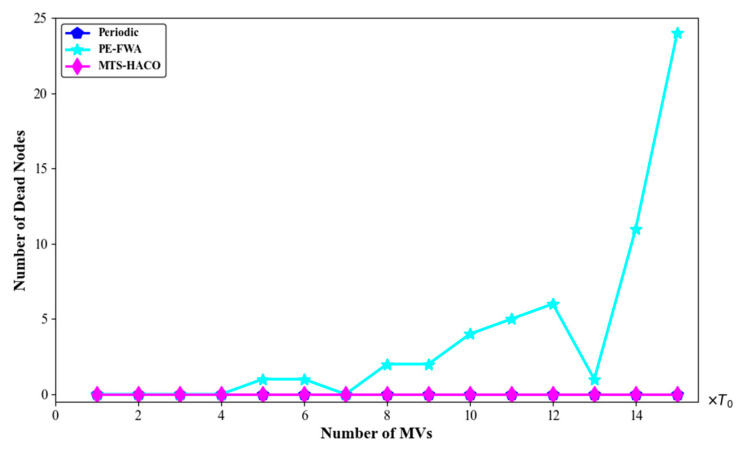
Dead nodes for the three algorithms.

**Figure 19:**
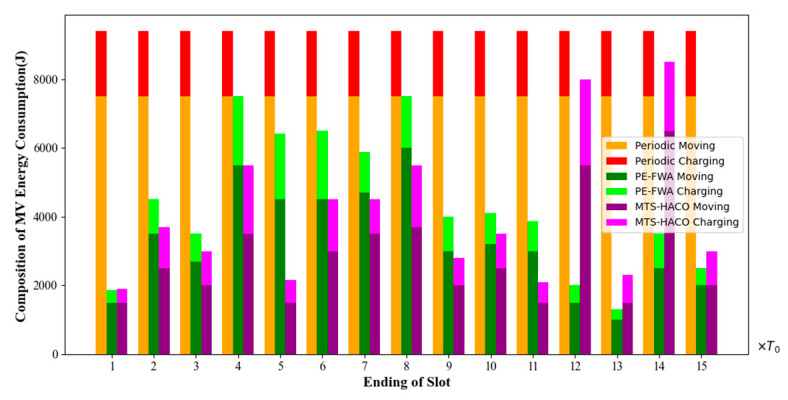
Energy consumption composition for the three algorithms.

**Figure 20:**
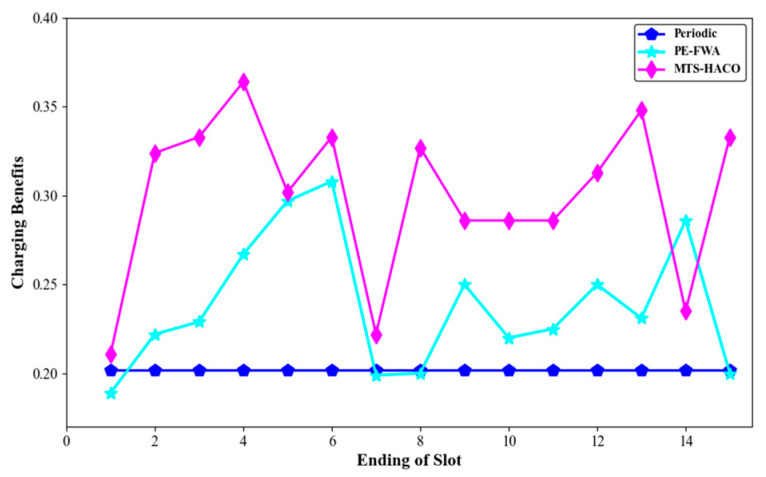
Charging benefits of the three algorithms.

**Figure 21:**
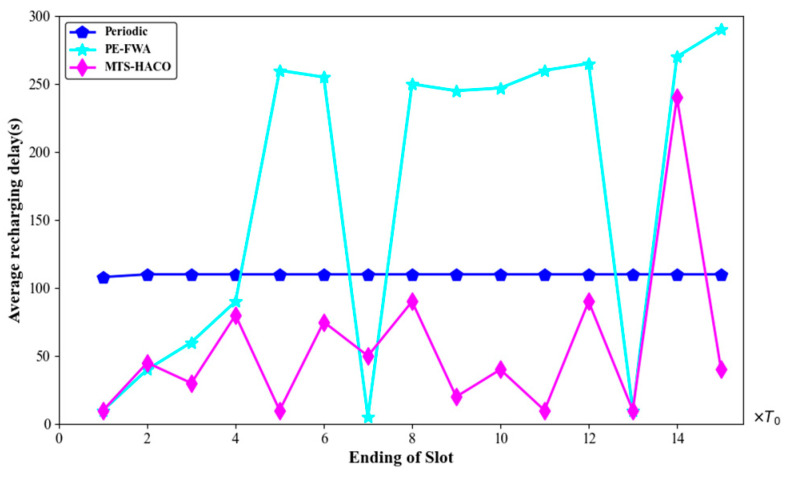
Average recharging delay with the three algorithms.

Figure 18, Figure 19, Figure 20 and Figure 21 present a comparison of the dead nodes, total MV energy consumption, charging benefit, and charging delay under the three charging strategies over one cycle.

As shown in Figure 18, under the PE-FWA strategy, in the early stages of network operation, the MV had sufficient energy and time to charge all the requesting nodes. However, as more nodes’ remaining energy dropped below the threshold, the number of cluster heads sending charging requests increased significantly, leading to a situation where the MV could not service all the cluster heads, resulting in an increasing number of node deaths. In contrast, under the periodic charging strategy and MTS-HACO, no nodes died throughout the entire cycle. As previously mentioned, the periodic charging strategy ensured that each node had enough power to survive until the next charging round by periodically charging all the nodes in the network. Therefore, no dead nodes occurred. Meanwhile, MTS-HACO established an independent energy replenishment cycle for each node, ensuring they were recharged before their energy was depleted. Consequently, there were no dead nodes, and the number of charging instances was reduced, improving the energy utilization of the MV.

Figure 19 shows the energy consumption composition of the MV in each charging time slot. It was evident that under the periodic charging strategy, the total energy consumption of the MV remained relatively stable and was significantly higher than that of the other two methods. This is because the periodic charging strategy resulted in longer travel distances for the MV. The average proportion of energy consumed for charging under each of the three charging strategies was 20.2%, 23.8%, and 30.0%, respectively. In the MTS-HACO algorithm, the MV charged only nodes with low remaining energy, with reasonably allocated and optimized trajectories. Therefore, it achieved the highest charging efficiency. In the PE-FWA strategy, the MV frequently moved back and forth between different cluster heads, leading to higher movement energy consumption compared with the MTS-HACO.

As shown in Figure 20, the charging benefit of the MTS-HACO algorithm was found to be significantly higher than that of the other two algorithms. It is important to note that in the 14th time slot of MTS-HACO, 17 nodes with low remaining energy sent charging requests, leading to substantial energy replenishment. As a result, in this time slot, the charging benefit of MTS-HACO was lower than that of the PE-FWA algorithm.

Figure 21 shows the average charging delay of the nodes; it is clear that MTS-HACO outperformed the other two charging strategies. Although there was a sudden increase in charging delay during the 14th time slot due to the sharp rise in the number of nodes sending charging requests, it still remained lower than with the PE-FWA strategy, with no node deaths.

In the periodic charging strategy, the fixed charging cycle and energy demand led to slight variations in charging delay within the cycle. In contrast, under the PE-FWA strategy, fewer nodes sent charging requests in the early stages of network operation, resulting in relatively short average charging delays, which were close to those of the MTS-HACO. However, as the number of nodes requiring charging increased, the average charging delay increased significantly. It is worth noting that the shorter delays in the 7th and 13th time slots were due to the smaller numbers of nodes needing charging.

## 7. Conclusions

Due to sensor node lifespans in precision agriculture and the impact of the MV’s travelling path on charging benefit, this paper proposes a mobile charging transition optimization algorithm based on the ant colony and fireworks algorithms. In this method, the remaining lifespan of nodes is used as a criterion for selecting charging nodes. We innovatively propose the Generalized Synchronizing Continuous Cyber Petri-net System, which unifies the energy flow, motion flow, and control flow in WRSNs. The algorithm’s performance was compared across different network topologies, and the simulation results indicate that, regardless of the network topology, the MTS-HACO algorithm demonstrated a significant advantage in terms of charging benefit. Furthermore, under various combinations of MV numbers and node counts, the MTS-HACO algorithm consistently exhibited superior performance.

In conclusion, the MTS-HACO algorithm can effectively improve the charging benefit of MVs while meeting the energy demands of the nodes and significantly reduce charging delays.

However, as the network scale continues to expand, this method may not be fully applicable. In real-world scenarios, factors such as terrain and obstacles impose additional demands on MVs’ scheduling. For instance, MVs may struggle to navigate rough and uneven mountainous terrain. Additionally, avoiding obstacles while reaching target nodes with minimal cost presents another challenge. Therefore, future work will focus on enhancing the algorithm’s adaptability to these practical conditions and scaling issues. Moreover, this approach can be further extended to other types of sensor networks. For example, in smart city applications, sensors are used to monitor traffic flow, air quality, or water resource management, where optimizing energy scheduling is equally crucial. Additionally, the field of environmental monitoring can also benefit from the charging scheduling strategies proposed in this study, especially in remote areas where improving node lifespan through optimized charging paths and energy allocation can significantly enhance data collection efficiency. Future research can explore how this method can be applied to other IoT scenarios with high energy demands and distributed architectures, such as industrial IoT and smart grids.

## Figures and Tables

**Table 1:** Parameter definitions of network model.

Symbol	Definition	Unit
m	number of mobile vehicles	-
n	number of sensor nodes	-
δ	moving consumption of a mobile vehicle	J/s
U	charging power of a mobile vehicle	J/s
σ	charging ratio	
$P_{s_{i}}$	energy consumption of the *i*th sensor node	J/s
$d_{i-1,i}^{j}$	travelling distance from the *i* − 1th node to *i*th node of the *j*th mobile vehicle	m
*v*	speed of a mobile vehicle	m/s
$\tau_{i,i+1}^{j}$	moving time from the *i* − 1th node to *i*th node of the *j*th mobile vehicle	s
$\tau_{mov}^{j}$	total moving time of the *j*th mobile vehicle	s
$\tau_{vac}^{j}$	dwell time of the *j*th mobile vehicle at base station	s
$\tau_{char}^{j}$	charging time of the *j*th mobile vehicle	s
$T_{0}$	duration of a slot	s

**Table 2:** Description of components of GSCCPNS.

Symbol	Definition	Category
$s_{i}$	energy of the *i*th sensor node	Place
$c_{j,i}$	energy of the *j*th mobile vehicle located at the *i*th sensor node	Place
$tr_{i-1,i}^{j}$	travelling event between two nodes of the *j*th mobile vehicle	Transition
$ch_{i}^{j}$	charging event of the *j*th mobile vehicle for the *i*th sensor node	Transition
$con_{s_{i}}$	consuming event of the *i*th sensor node	Transition
$W\left(tr_{i-1,i}^{j},c_{j,i}\right)$	moving consumption of the *j*th mobile vehicle	Weight
$W\left(c_{j,i},ch_{i}^{j}\right)$	charging power of the *j*th mobile vehicle	Weight
$W\left(ch_{i}^{j},s_{i}\right)$	receiving power of the *i*th sensor node	Weight
$W\left(s_{i},con_{s_{i}}\right)$	consuming power of the *i*th sensor node	Weight

## Data Availability

The data presented in this study are available on request from the corresponding author. The data are not publicly available due to privacy.
